# A qualitative systematic review of barriers and facilitators to the implementation of community-based molecular diagnostics for infectious diseases

**DOI:** 10.1371/journal.pone.0321690

**Published:** 2025-05-13

**Authors:** Hannah Nelson, Jia Tong Song, Mai-Lei Woo Kinshella, Jennifer Cochrane, Karen Mooder, Kasra Hassani, Michelle Dittrick, David M. Goldfarb

**Affiliations:** 1 Experimental Medicine Graduate Program, University of British Columbia, Vancouver, British Columbia, Canada; 2 Department of Pathology and Laboratory Medicine, BC Children’s and Women’s Hospital & Health Centre, Vancouver, British Columbia, Canada; 3 Department of Medicine, University of British Columbia, Vancouver, British Columbia, Canada; 4 BC Children’s Hospital Research Institute, Vancouver, British Columbia, Canada; 5 Department of Obstetrics and Gynecology, University of British Columbia, Vancouver, British Columbia, Canada; 6 Community Based Testing and Biomedical Initiatives, First Nations Health Authority, Vancouver, British Columbia, Canada; 7 First Nations Health Authority, Vancouver, British Columbia, Canada; 8 Department of Pathology and Laboratory Medicine, University of British Columbia, Vancouver, British Columbia, Canada; Stellenbosch University, SOUTH AFRICA

## Abstract

**Background:**

Community-based molecular diagnostic testing for infectious diseases can bring equitable healthcare to resource-limited settings without hospital-based laboratories. Low-complexity molecular testing devices allow for unprecedented sensitivity and specificity for infectious disease diagnostics outside of a dedicated laboratory, which may facilitate timely initiation of therapeutics and other public health interventions in rural, remote and/or marginalized communities.

**Objective:**

To identify barriers and facilitators to the implementation of community-based molecular testing.

**Methods:**

A systematic search was conducted on MEDLINE Ovid, EMBASE, Web of Science, Cumulative Index to Nursing and Allied Health, Google Scholar, and reference lists. Original research that includes implementation of molecular testing systems at the community-level or at rural/remote health facilities with frontline healthcare workers and reports barriers and facilitators to implementation were included. Studies were assessed by the Critical Appraisal Skills Programme Qualitative Checklist and underwent inductive thematic analysis. The review protocol was registered to Prospero prior to conducting the review (CRD42023397800).

**Results:**

A total of 6 studies were included in the review. We found three main themes present across all six included studies: infrastructure, usability, and staffing considerations. Infrastructure emerged as a critical determinant, with challenges ranging from physical space constraints to issues with reliable electricity and internet connectivity. The usability of testing devices, encompassing factors like ease of use and testing quality, also played a pivotal role. Staffing considerations, including workload, training, and attitudes, significantly influenced implementation outcomes.

**Conclusion:**

Our review highlights the importance of addressing infrastructural challenges, ensuring usability of testing devices, and adequately supporting staff through training and workload management to realize the full potential of this new opportunity. Future implementation should consider these factors to successfully integrate molecular diagnostics into community-level healthcare delivery, particularly in rural and remote areas.

## Introduction

Community-based molecular diagnostic testing for infectious diseases has the potential to reduce diagnostic delays, improve public health management, and allow for rational antimicrobial utilization, and therefore may increase access to equitable healthcare in resource-limited settings which lack hospital-based laboratories. A key marker of point of care (POC) testing across multiple definitions is rapid turnaround time to allow for quick diagnosis, and therefore rapid referral, public health interventions, and/or treatment decisions [1–4]. At the community-level, quick testing may facilitate timely initiation of therapeutics and other clinical and public health interventions that would otherwise be delayed if there is a need to wait for return of laboratory-based test results [5,6]. These delays may be significantly intensified in rural, remote or resource-constrained settings [7] such as First Nation communities. Evidence of successful POC testing in rural and remote communities can be found in publications from Australia, where a variety of tests have been implemented at the POC in remote Indigenous communities utilizing various technologies [8–10].

Low complexity molecular testing refers to molecular testing systems where no special infrastructure or laboratory skills are required to run the test and interpret the results. While low complexity, lower quality (low sensitivity/specificity) POC tests have been used for many years in these settings, further advances in access to low complexity, higher quality (high sensitivity/specificity) molecular diagnostics in non-laboratory settings have been made particularly in the context of the COVID-19 pandemic [4,11,12]. This progress includes the increased utilization of molecular testing with low complexity devices outside of dedicated laboratories [13]. Accessing molecular diagnostics with these devices may allow for unprecedented sensitivity and specificity when making a diagnosis of an infectious disease in communities [12–14]. As such, we define community-based testing as high-performance testing that may be used to inform clinical decision making without additional confirmation. However, establishing and maintaining testing pathways present significant challenges. While literature exists on implementation factors for molecular and non-molecular POC testing [6,15,16], there is a current gap in the literature regarding reviews of barriers and facilitators to implementation of molecular diagnostics in community based settings, outside of an accredited laboratory. This highlights the need to explore studies that have already implemented molecular diagnostics at the community-level and identify what key factors contributed to successful implementation.

As such, this review aims to understand barriers and facilitators to implementation of community-based testing to inform future implementation of testing for different infectious diseases and promote optimized patient care for people residing in rural or remote areas with no hospital-based laboratory. This review also aims to reflect on its findings regarding the closing of diagnostic service gaps between First Nation and non-First Nation peoples caused by geographic barriers, systemic racism, and colonial policies and practices that uphold health inequities.

## Methods

The objective of this review is to examine barriers and facilitators to implementation of community-based or point of care molecular testing. Community-based settings were defined as settings lacking a hospital-based laboratory to encompass testing in communities that may not be defined as true POC testing. Review methods were developed in accordance with the Preferred Reporting Items for Systematic Reviews and Meta-analyses (PRISMA) checklist (S1 Table) [17]. A review protocol was developed and registered to Prospero (CRD42023397800).

### Search strategy

Searches were conducted on MEDLINE, Embase, Web of Science, Cumulative Index to Nursing and Allied Health (CINAHL), World Health Organization database and Google Scholar from database conception until February 2024, with no limits applied on date of publication or language. The last search date for all sources was February 5^th^, 2024. Searches were supplemented by scanning reference lists of included studies. Search terms were based off the PICOS (Population, Intervention, Comparison, Outcome, and Study) framework (Table 1), and included search terms point of care, community-based testing, low resource setting, molecular testing, GeneXpert, PCR systems, antiviral (S2 Table).

**Table 1:** PICOS review framework.

	Inclusion	Exclude
**P**opulation	Frontline healthcare workers and community stakeholders	Lab-technologists based in a hospital-laboratory
**I**ntervention	Implementation of community-based or point of care molecular testing	Molecular testing only a hospital- based laboratory or reports of only non-molecular point of care testing
**C**omparison	No intervention, hospital, or laboratory-based molecular testing	n/a
**O**utcome	Barriers and facilitators to implementation of community-based or point of care molecular diagnostics	Did not report barriers and facilitators to implementation
**S**tudy	Experimental studies (controlled trials) and observational studies (cohort, case controlled, cross-sectional, qualitative)	Reports lacking full methodological details

To acknowledge other ways of knowing aside from the traditional western academic ideology, we focused on broadening our search to be more inclusive of other forms of knowledge and practice, and emphasizing a reflexive process at each stage of the review [18]. This was accomplished through searching grey literature, examining non-peer reviewed content, and having discussions among the study team on how to evaluate and screen reports. There are different ways of communicating/describing evidence, and the populations for which community based testing interventions are optimally suited soften bring more diverse worldviews and methods of recording scientific evidence [18]. This methodology was used to try to find First Nation or Indigenous perspectives which may not be published in traditional academic literature. We have also undertaken reflexivity to consider the perspectives and biases that we as researchers carry, and attempted to conduct this research with community applicability in mind. In our case, this would be taking what we learn in this review to inform future implementation of testing in communities, and ensuring that the information is disseminated in an accessible format.

### Inclusion and exclusion criteria

Studies were screened for inclusion if they implemented molecular testing systems at the community-level or at health facilities with frontline clinical health workers. This included both POC and near-POC testing. Secondary-level hospitals, including regional, referral, district and rural hospitals were considered if they lacked a designated laboratory with professional lab technologists. Studies including only molecular testing via professional laboratory staff at a hospital-based laboratory were excluded, however studies were still screened for inclusion if they displayed implementation for both point of care and urban settings. Our outcome of interest was barriers and facilitators to implementing community-based or point of care molecular testing, therefore studies were excluded if they did not report a qualitative component that yielded such outcomes. To assess implementation factors, we excluded publications that lacked primary results or included participants who did not have experience with implementation such as review articles, feasibility studies, commentaries, and protocols. Studies were not excluded based on study design, with the exception of letter to the editors, conference abstracts and other publications without full methodological reporting. Lastly, publications were excluded if they were not available in English.

### Study selection

All title and abstracts were screened by two independent reviewers (HN and JS) according to the inclusion and exclusion criteria. Any discrepancies were resolved with discussion and in the absence of a consensus a third reviewer (MWK) was asked to adjudicate. Next, full texts were subject to independent review by two reviewers (HN and JS). A third reviewer (MWK) was available for adjudication when necessary.

### Quality assessment

Two reviewers (HN and JS) independently completed a quality assessment for each included study. The Critical Appraisal Skills Programme (CASP) tool was used to assess study quality due to its prevalence in health-related qualitative evidence synthesis [19,20]. Studies were not excluded as a result of the quality assessment to ensure comprehensive identification of the barriers and/or facilitators to implementation.

### Data extraction and analysis

A data extraction sheet was developed by the study team and refined by two reviewers (HN and JS). One reviewer (HN) extracted all demographic and methodological data in the sheet including qualitative methodology, sample size, participant roles, country, facility type, molecular testing device, and molecular testing implemented. Full text of each included study was imported into NVivo 14 (QSR International, Melbourne, Australia) where they were analyzed using an inductive thematic approach to identify barriers and facilitators to implementation [21]. Following familiarization with the studies, two reviewers (HN and JS) collaboratively used the excerpts to develop codes that denote key themes, which were then separated in the subthemes. Each study was then independently analyzed by the two reviewers (HN and JS).

## Results

### Characteristics of included studies

**Fig 1:**
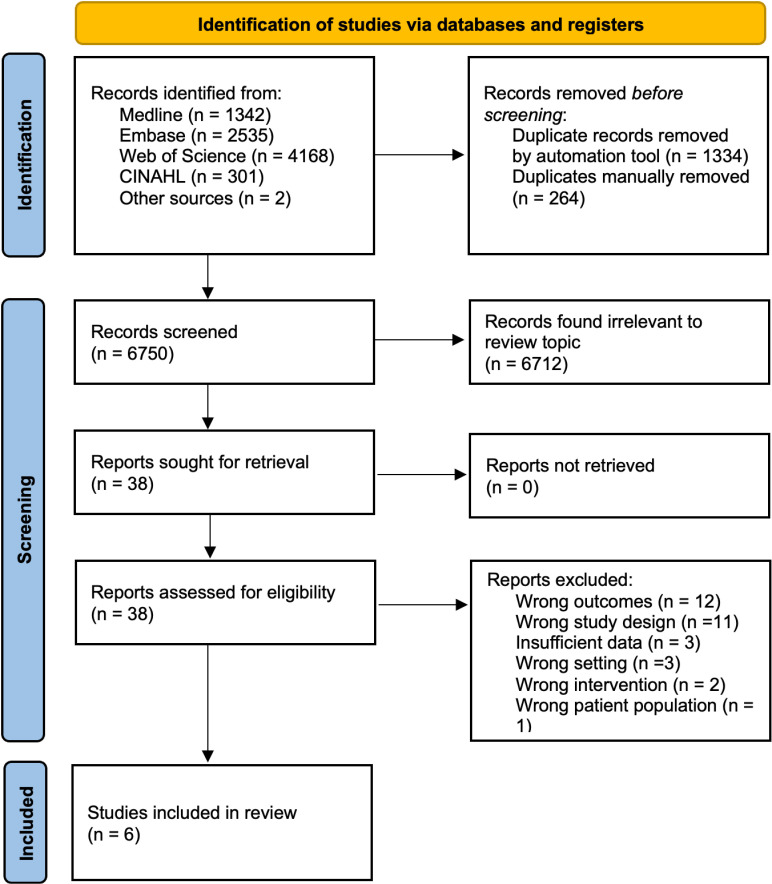
PRISMA diagram of included and excluded studies.

We identified a total of 6750 articles to screen after the removal of duplicates (Fig 1). Of that, 6712 records were excluded based on the inclusion/exclusion criteria and 38 full texts were assessed for eligibility. Reasons for exclusion were no qualitative outcomes (n = 12), lack of community-based testing implementation or experience (n = 11), wrong setting (n = 3), abstract or report lacking sufficient methodological descriptions (n = 3), wrong intervention (n = 2), and wrong patient population (n = 1) (S4 Table).

Of the 6 included studies, all were published between 2015 and 2022 (S5 Table). There was one multicounty study that was conducted in 18 low and middle income countries (LMICs) supported by Médecins Sans Frontières [22]. Other single country studies were conducted in Zimbabwe [23], Papa New Guinea [24], Kenya [25], South Africa [26], and Australia [27] (Table 2). The study conducted in Australia was carried out in remote Aboriginal communities.

**Table 2:** Characteristics of included studies.

Reference	Country	Facility type	Qualitative data collection method	Participant description	Total Sample size	Participant distribution	Testing implemented
Martin et al. 2022	Zimbabwe	Community-care centre	In-depth interviews	Program (CHIEDZA) providers (community health workers (CHWs), nurses, youth workers, a counsellor) and team members involved in the development and co-ordination of on-site testing (the trial co-ordinator, a microbiologist, and a laboratory technician)	12	CHWs = 4 Nurses = 2 Youth workers = 2 Counsellor = 1 Development team members = 3	Xpert® CT/NG
Mohammad et al. 2020	Papua New Guinea	Two provincial hospitals with no laboratory technicians	Semi-structured interviews	Nurses and key informants (senior prevention of mother-to-child transmission [PMTCT] programme staff, Central Public Health Laboratory staff, national government representatives, and NGO staff)	18	Nurses = 5 Programme staff = 5 Laboratory staff = 2 Government representatives = 3 NGO staff = 3	Xpert® HIV-1 qualitative
Opollo et al. 2018	Kenya	Sub-county hospital (1), health centers (3) and dispensaries (22) for sample collection, with central hubs between them for testing (4)	Self-administered questionnaire	Laboratory technologists conducting testing in the field	Not reported	Not reported	Xpert® HIV-1
Ardizzoni et al. 2015	18 LMICs*	District and sub-district laboratories (21), regional facilities (5), peripheral facilities (6) and one penitentiary system facility	Questionnaire	Site laboratory coordinators	28	Laboratory coordinators = 28	Xpert® MTB/RIF
Engel et al. 2015	South Africa	Urban and rural public or private settings, ranging from community/clinic setting to hospital or peripheral lab	Semi-structured interviews	Doctors, nurses, CHWs, patients, laboratory technicians, policymakers, hospital managers and diagnostic manufacturers	101	Not reported	Variety of tests for HIV TB including Xpert® MTB/RIF
			Focus group discussions	TB patients, nurses, and community health workers	40 (7 groups)		
Natoli et al. 2015	Australia	Aboriginal community-controlled health services in remote communities (12)	In-depth interviews	Registered or enrolled nurses and Aboriginal Health Workers/Practitioners	16		Xpert® CT/NG

*Cambodia, Central African Republic, Colombia, Democratic Republic of Congo, Georgia, India, Kenya, Kyrgyzstan, Lesotho, Malawi, Mozambique, Myanmar, Russia, Somalia, South Africa, Swaziland, Uzbekistan, Zimbabwe

Three of the studies conducted interviews [23,24,27], one study conducted both interviews and focus group discussions [26], and two studies utilized questionnaires with some open-ended questions [22,25] (Table 2). The study populations included but were not limited to community-health workers (CHWs) [23,26,27], nurses [23,24,26,27], community stakeholders [23,24,26], research program staff [22–24], and laboratory technicians [23–26] (Table 2). All six studies conducted at least a portion of testing using a GeneXpert® assay; two studies implemented the Xpert**®** CT/NG assay for detection of *Chlamydia* and *Gonorrhea* [23,27], two studies implemented the Xpert® HIV-1 qualitative assay for early infant diagnosis (EID) of *human immunodeficiency virus* (HIV)[24,25], and two studies implemented or spoke to healthcare workers with experience using the Xpert® MTB/RIF for detection of *Mycobacterium tuberculosis* (TB) complex and Rifampin resistance-associated mutations [22,26].

### Quality assessment

Based on the CASP checklist, all included studies ranked ‘good’. Three studies included all CASP checklist components and three studies omitted one component, namely, consideration of relationships between the researcher and the participants [22,25,26]. The full breakdown of results for the CASP checklist can be found in S3 Table.

### Themes

We found three main emergent themes present across all six included studies: infrastructure, usability, and staffing considerations. Infrastructure included physical space, reliable electricity and internet connection, temperature regulations, and consumables subthemes. Subthemes for usability included ease of use, device limitations, and testing quality. Subthemes for staffing included workload, training, and attitudes. Minor themes present in three or more studies included community acceptance and impact on treatment. Main and minor themes are detailed below and reported in Table 3.

**Table 3:** Emergent themes and subthemes for included studies.

	Themes	Sub-themes
Main themes	Infrastructure	- Physical space - Reliable electricity and internet connection - Temperature regulation - Consumables
	Usability	- Ease of use - Device limitations - Testing quality
	Staff	- Workload - Training - Attitudes
Minor themes	Community acceptance	
	Impact on treatment	

#### Infrastructure.

All six studies mentioned challenges with inadequate infrastructure [22–27]. This included difficulty provisioning physical space for testing, maintaining sufficient internet connection and electricity, setting up air conditioning for temperature regulation, and maintaining a supply of consumable test cartridges. The implementation of the molecular devices required space to place the machine and test materials, which led to either infrastructure renovations [22] or various solutions to make space in the existing site infrastructure. An example excerpt from Mohammed et al. described challenges with physical space: “*Both study sites have a high patient load and limited space, and health workers highlighted the challenges of trying to fit the new technology into the clinic*” [24]. One study described a community-site that provided services in an outside space, where the GeneXpert**®** device was placed in a booth that was also used for clinical consultations and led to delays in sample testing when the booth was in use [23]. Further infrastructure barriers were the limited internet and electricity access across some sites [22,23,25,26]. A participant elaborated: *“… not all the clinics have good Internet or signal”*[26]. Unstable connections led to issues with device calibration, error codes from interrupted power supply, and failed sample testing which needed to be redone. Strategies to mitigate power loss included utilizing batteries or generators as a backup, though this presented further concerns with changing power supply. Barriers to temperature regulation for the testing device and kits were noted. Ardizzoni et al. mentioned for GeneXpert® MTB/RIF testing: *“…implementation required costly interventions, including provision of air conditioning…”* [23]. Lastly, a study in Papa New Guinea highlighted the importance of maintaining a sufficient supply of consumables such as test kits: “*Ensuring a sustainable and consistent supply of test cartridges was also identified as an important aspect of integrating POC EID testing into routine clinical care.”* [24]

#### Usability.

Usability was discussed in all six studies, including the testing device’s ease of use, device limitations, and testing quality [22–27]. Some studies reported testing with the GeneXpert® as straightforward and easy to use [22,25], as demonstrated by the following excerpt: “*Overally the staff reported that the GeneXpert was a high performing device that was easy and feasible to use within the field setting”* [25]*.* Other studies expressed difficulty with how sensitive the machine was to mistakes [23], as well as concerns over the need for regular maintenance [24], and reported challenges with sample preparation [27]. Insufficient testing capacity (with either a two or four module device) for the number of samples collected each day [23], lack of available assays for some infectious diseases [27], and high error or inconclusive result rate depending on the device operator [22] were also reported as limitations of the testing device. A multicounty study where the tests were run by a variety of staff reported, *“High rates of inconclusive results represented one of the major challenges, related to various factors including technical issues and staff experience”* [22]*.* Inconclusive results impacted the perceived quality of testing in some participants [22,27], and aligned with concern about quality outside of a laboratory setting [24–26]. A health professional from the Papa New Guinea study highlighted, *“…because it’s a machine that is being used in the lab, [when] we try to take it out from the lab, put it in the clinic… [the] challenge is that the quality and the results being produced because there are likely to be mistakes…”*

#### Staffing considerations.

Staffing considerations, including workload, training, or attitudes, were described in all six studies [22–27]. Increased staff workload was reported as a barrier to implementation in two studies [23,26]. Staff were expected to conduct testing in addition to their existing duties, resulting in a backlog of tests in busy settings. Providers sometimes artificially prolonged turnaround times to mitigate the increase in workload [23]. A program worker in Zimbabwe remarked, “*[W]e had more work to [do] … we needed to allocate at least one person to be running the GeneXpert machine … [which] meant that we would one or two people less for the day in the [HIV program] booths*” [23]. Another study highlighted that while research nurses facilitated implementation, integrating EID HIV testing into their existing workload would have been challenging [24]. However, some health workers interviewed in the same study suggested that POC EID testing would reduce workload by alleviating tasks related to shipment of samples for testing and the time spent waiting for results [24]. Adequate training with the molecular testing platform was a key facilitator described [22–24]. Providers described running the device as *“simple and straightforward”* following training, and noted that they became more confident using the device over time [23]. There was emphasis on continuous training and ongoing technical support, especially for those with no prior experience in laboratory diagnostics [22]. The amount of training and experience positively impacted staff attitudes towards testing following implementation, as illustrated in an excerpt from the Zimbabwe study: *“…providers noted that they did find using the machine difficult initially, however with experience they became more confident in running tests*” [23]. Additionally, there was evidence of staff empowerment at being able to test and treat on-site, as well as increased awareness of some diseases being tested for including sexually transmitted infections (STIs)[27]. Positive attitudes and perceived importance towards testing made the increases in workload more acceptable for some healthcare workers in Papua New Guinea [24].

#### Minor themes.

Minor themes reported in at least three studies included community acceptance and impact on individual treatment, which varied across studies. Levels of community acceptance varied from good acceptability in Australia with STI testing [27], to reluctance to wait for results among youth in Zimbabwe getting STI testing [23] and among families with babies undergoing EID HIV testing [24]. An excerpt from Mohamed et al. remarked: *“POC testing was not able to address competing priorities that made it difficult for caregivers to stay at the clinic”*[24]*.*

The ability to impact treatment was described as a facilitator. The ability to commence antiviral treatment ‘straight away’ on HIV positive infants was described as an advantage of POC testing [24]. Additionally, the reduction in time to treat was also stated as particularly important for remote communities in Australia [27], as demonstrated by the following excerpt: “*For many participants, one of the overriding advantages of point-of-care testing was reducing the time between specimen collection and treatment provision.”*

## Discussion

This review focused on barriers and facilitators to the implementation of community-based molecular testing for infectious disease pathogens (S1 Fig). In our analysis, we found having a sufficient number of staff to run the testing devices was an important facilitator to the success of POC programs, and the training they received positively influenced attitudes towards testing. Further facilitators included the easy-to-use testing devices and staff empowerment in testing. Significant barriers related to maintaining adequate infrastructure for testing, including both physical space and electrical connections. While the testing devices were typically reported as easy to use, there were some frustrations with inconclusive test results and perceived poor quality of testing. Preparing for, and addressing these barriers while uplifting facilitators is critical in maintaining confidence for molecular testing programs in communities.

The disruption to clinical workflow arising from stringency of available health resources was an overarching theme observed throughout this review. Implementation of testing led to delays in clinical processes, and directly impacted the turnaround time for results and continuity of established workflow. Frequent inconclusive results delayed workflow [22,23] and increased result turnaround time when a sample needed to be recollected. Multiple sites did not provide timely testing in communities adjacent to clinical encounters, and instead opted to call patients with results later in the day or in days following sample collection [22,23,26,27]. Suggestions to streamline the workflow included better connectivity of the GeneXpert to patient care (clinical informatics) systems to alleviate the burden of having to manually enter results [27]. Increased resources may also provide more opportunity to dedicate staff towards this workflow and decrease workflow challenges.

All included studies were published after 2015 and all but one studied implementation in LMICs. This is in line with increasing demand and delivery of all POC and near POC testing over the years, as well as a response to demand for low complexity and fast diagnostic devices in resource-limited settings. We begin to observe publications following the development of low complexity molecular testing devices and the validation of assays such as the Xpert**®** MTB/RIF in 2004, which then went on to be endorsed by the World Health Organization for initial TB testing in 2011 [28,29].

Each included study was found to be “good” with a quality assessment tool, but there was a low number of studies due to a lack of publications with qualitative methodology exploring implementation factors. The strength of the evidence is support by the research quality and identification of common themes throughout the six included studies, but the low number of studies may indicate missing bodies of knowledge. Reasons for this may include a lack of focus of community voices and bias arising from constraining systematic reviews to westernized approaches to presenting evidence and information. Further, this missing knowledge may consist of other studies evaluating feasibility prior to implementation, or molecular testing programs that only shared implementation data presented in a quantitative comparison of diagnostic assays.

Another systematic review examined the feasibility and value of implementation of molecular chlamydia and gonorrhea (GeneXpert**®** CT/NG) POC testing into routine practice[30]. The authors developed a unique value proposition to facilitate decision-making around integration of testing into healthcare, and demonstrated its use in different settings. It highlights the considerations needed when implementing CT/NG POC testing across different settings, and guides stakeholders to make POC testing choices based on their site complexities. By integrating both lessons learned from feasibility studies and implementation perspectives presented in our results, community-based testing programs may be more successful and sustainable long term.

Interestingly, community acceptance was explored more in studies that implemented POC STI diagnostics [23,24,27], whereas we noted a gap in the literature with testing for TB. When community-acceptance was considered, it appeared these tests were of lower prioritization to the community, and that people from the community had competing interests that made them less willing to wait for test results for TB. This highlights the need to engage communities and receive their wisdom about their own health priorities and concerns about harm or stigma around adopting certain types of diagnostics.

A study published in the United States exploring the barriers and facilitators to non-molecular point of care tests in family medicine clinics presented similar results [31]. This study found similar themes such as the impact of testing on staff and clinic workflow, as well as the impact on clinical decision making. Reported barriers to testing were concerns that increased testing volume may extend patient visits or overwhelm providers, and that there may be insufficient staff within clinics to manage additional testing [31]. Facilitators included easier patient follow-up for laboratory tests between office visits, less testing strain shifted to under-staffed laboratories, faster decision-making, earlier triaging of possible serious illness, and improved confidence in treatment decisions [31]. The overlap with our reported themes supports that many implementation factors for POC testing are translatable to non-molecular testing, as well as to other settings in high income countries such as the United States.

Another study implemented molecular testing for *human papillomavirus* (HPV) in Kenya, utilizing the GeneXpert platforms already implemented in county and sub-county hospitals [32]. In this pilot study, cervical samples were either self-collected or collected by CHWs in communities, then transported to the testing facilities. The authors used the nominal group technique to identify that self-sampling was a more acceptable collection method, and that having CHWs go into communities for sampling increased screening uptake. Some challenges were noted to be low community awareness, poor linkage and communication between clinicians and laboratory personnel that ran the HPV tests leading to delays, high loss of follow-up, and low retention of trained staff at screening sites [32]. When considering the barriers we identified with inconclusive results and concerns with quality outside of the laboratory, having CHWs go into communities to collect samples to bring back to laboratory personnel for testing presents a potential solution. However, this may not be feasible in all settings and presents certain complexities related to interfacing between the clinical setting and laboratory. Regardless, this study presents further evidence that community-based approaches are successful, and that there may be benefits to exploring self-sampling for sample collection for testing in other settings.

### Strengths and limitations

Strengths of this study include the rigorous methodology and sound study design, as outlined by the PRISMA guidelines, as well as the novel results. To our knowledge, no other review has been published on the barriers and facilitators to implementation of community-based molecular diagnostic testing. We provide a unique synthesis of qualitative evidence following implementation of testing and we aim to influence future molecular diagnostic initiatives at the community level by providing areas to consider when creating these complex programs. Community-based molecular testing has potential to strengthen health systems and optimize patient care in rural, remote, First Nation and other underserved communities. To work towards successful and sustained implementation, site specific considerations need to be accounted for prior to implementation to prevent further overburdening of staff working in such settings. Future research may explore factors that determine long-term success and sustainability of POC or near POC molecular testing initiatives.

In some studies, the qualitative data was inclusive of both rural and urban setting, with little differentiation available in the results. In these cases, the studies were still included if there was a portion of rural or remote data, with the results being carefully analyzed to try and isolate findings from rural areas. Many of the barriers and facilitators are shared across locations and facility type, such as testing device training or machine usability. Similarly, one study focused on all POC testing and included sites that used molecular diagnostics for TB testing, but also a variety of other POC tests[26]. This study was still included as it met the inclusion criteria for participants with experience in community-based testing implementation, but we acknowledge that isolating qualitative results specific to both molecular testing and remote areas was not always possible. In addition, our search yielded a small number of studies due to the focus on implementation factors and qualitative data. Several informative prospective qualitative studies were evaluated but ultimately excluded due to the lack of participant experience with community-based testing.

There was also notable gap in the literature regarding First Nations specific studies, despite methodological steps to seek them out. This included a lack of both reports on molecular diagnostic testing for infectious diseases in First Nations communities, as well studies examining systemic racism in infectious disease diagnostics. There is a need to implement and uplift work that addresses the diagnostic service barriers faced by First Nations peoples, as well as consider the systemic factors that contribute to health inequities such as legislation and regulation that constrain access. Future research should take into consideration the colonial policies and practices that have led to health inequities, and consider implications for their local First Nations or Indigenous communities.

## Conclusion

The implementation of community-based molecular diagnostic testing for infectious diseases holds significant promise for enhancing healthcare delivery, particularly in resource-limited settings. In First Nation communities, implementation of community-based testing programs could address diagnostic service gaps and contribute to more equitable healthcare and improved health outcomes. Through a qualitative systematic review of six included studies, we identified key barriers and facilitators shaping the success of such novel initiatives. Infrastructure emerged as a critical determinate in implementing molecular diagnostic devices, highlighting challenges with limited physical space, unreliable internet and electricity, and the need for temperature regulation. Ensuring a consistent supply of consumables was also essential to support sustainable testing in resource-limited settings. The usability of molecular testing devices such as the GeneXpert® also played a pivotal role. The GeneXpert® testing device was generally user-friendly, though some studies highlighted limitations such as its sensitivity to user errors, frequent maintenance needs, and insufficient testing capacity. High rates of inconclusive results, influenced by technical issues and staff experience, raised concerns about testing quality outside laboratory environments. With regards to staffing considerations, the increased staff workload from implementing the testing posed challenges, but adequate training and support helped staff become confident with its use. Although the added workload was initially a barrier, positive attitudes and the perceived importance of on-site testing made it more acceptable for some healthcare workers.

Our findings underscore the importance of addressing multifaceted challenges to ensure successful and sustained implementation. Strategies aimed at overcoming infrastructure limitations, such as investing in adequate physical space and robust connectivity infrastructure, are crucial. Comprehensive training programs and ongoing technical support for frontline healthcare workers are also essential to optimize testing performance and mitigate potential barriers arising from increased staff workload and attitudinal issues that may arise. Overall, our review highlights the need for community-centered approaches that prioritize understanding and community acceptance, particularly in settings where competing priorities may impact willingness to engage with testing services.

## Supporting information

S1 Table PRISMA checklist. (DOCX)

S2 Table Search terms. (DOCX)

S3 Table CASP checklist for each study. (DOCX)

S4 Table Full-text study exclusion. (DOCX)

S5 Table Included studies data extraction. (DOCX)

S1 Fig Infographic of key findings. (PDF)
