# Supplementary material for: A qualitative systematic review of barriers and facilitators to the implementation of community-based molecular diagnostics for infectious diseases
Source: PLoS One. 2025 May 13;20(5):e0321690. doi: 10.1371/journal.pone.0321690 (PMC12074526; doi:10.1371/journal.pone.0321690)
Supplement: S2 Table — (DOCX) [file pone.0321690.s002.docx]

S2 Table. Search terms

| 1 | point-of-care/ |
| --- | --- |
| 2 | community-based testing or rural or remote or low resource setting or point-of-care or “point-of-care” |
| 3 | 1 or 2 |
| 4 | Xpert or GeneExpert or IDNow or PCR Systems or Antiviral or molecular testing |
| 5 | 3 and 4 |
