# Supplementary material for: A qualitative systematic review of barriers and facilitators to the implementation of community-based molecular diagnostics for infectious diseases
Source: PLoS One. 2025 May 13;20(5):e0321690. doi: 10.1371/journal.pone.0321690 (PMC12074526; doi:10.1371/journal.pone.0321690)
Supplement: S3 Table — (DOCX) [file pone.0321690.s003.docx]

S3 Table. CASP checklist for each study

| **Author and Year** | **1** | **2** | **3** | **4** | **5** | **6** | **7** | **8** | **9** | **Quality** |
| --- | --- | --- | --- | --- | --- | --- | --- | --- | --- | --- |
| Martin et al. 2022 | Y | Y | Y | Y | Y | Y | Y | Y | Y | Good |
| Mohammad et al. 2020 | Y | Y | Y | Y | Y | Y | Y | Y | Y | Good |
| Opollo et al. 2018 | Y | Y | Y | Y | Y | N | Y | Y | Y | Good |
| Ardizzoni et al. 2015 | Y | Y | Y | Y | Y | N | Y | Y | Y | Good |
| Engel et al. 2015 | Y | Y | Y | Y | Y | N | Y | Y | Y | Good |
| Natoli et al. 2015 | Y | Y | Y | Y | Y | Y | Y | Y | Y | Good |
