# Supplementary material for: A qualitative systematic review of barriers and facilitators to the implementation of community-based molecular diagnostics for infectious diseases
Source: PLoS One. 2025 May 13;20(5):e0321690. doi: 10.1371/journal.pone.0321690 (PMC12074526; doi:10.1371/journal.pone.0321690)
Supplement: S4 Table — (DOCX) [file pone.0321690.s004.docx]

S4 Table. Full-text study exclusion

| Number | Reference | Excluded On | Excluded By | Reason | Notes |
| --- | --- | --- | --- | --- | --- |
| 1 | Comparative evaluation of the diagnostic performance of the prototype cepheid GeneXpert ebola assay  Van Vuren P.J.; Grobbelaar A.; Storm N.; Conteh O.; Konneh K.; Kamara A.; Sanne I.; Paweska J.T.  J. Clin. Microbiol. 2016;54(2):359-367  2016  DOI: [10.1128/JCM.02724-15](https://dx.doi.org/10.1128/JCM.02724-15) | 24/05/23 | HN, JS | Wrong outcome | No qualitative component |
| 2 | The impact of Xpert (R) MTB/RIF depends on service coordination: experience in Burkina Faso  Moyenga, I; Roggi, A; Sulis, G; Diande, S; Tamboura, D; Tagliani, E; Castelli, F; Matteelli, A  INTERNATIONAL JOURNAL OF TUBERCULOSIS AND LUNG DISEASE 2015;19(3):285-287  2015  DOI: [10.5588/ijtld.14.0629](https://dx.doi.org/10.5588/ijtld.14.0629) | 24/05/23 | HN, JS | Wrong study design | No original research – just extrapolations from a database |
| 3 | Implementation of an in-house quantitative real-time polymerase chain reaction method for Hepatitis B virus quantification in West African countries  Ghosh S.; Sow A.; Guillot C.; Jeng A.; Ndow G.; Njie R.; Toure S.; Diop M.; Mboup S.; Kane C.T.; Lemoine M.; Thursz M.; Zoulim F.; Mendy M.; Chemin I.  J. Viral Hepatitis 2016;23(11):897-904  2016  DOI: [10.1111/jvh.12561](https://dx.doi.org/10.1111/jvh.12561) | 24/05/23 | HN, JS | Wrong outcome | No qualitative component |
| 4 | Evaluation of mobile real-time polymerase chain reaction tests for the detection of severe acute respiratory syndrome coronavirus 2  Onyilagha, C; Mistry, H; Marszal, P; Pinette, M; Kobasa, D; Tailor, N; Berhane, Y; Nfon, C; Pickering, B; Mubareka, S; Bulir, D; Chong, S; Kozak, R; Ambagala, A  SCIENTIFIC REPORTS 2021;11(1):  2021  DOI: [10.1038/s41598-021-88625-6](https://dx.doi.org/10.1038/s41598-021-88625-6) | 03/06/23 | HN, JS | Wrong outcome | No qualitative component |
| 5 | Do point-of-care tests (POCTs) offer a new paradigm for the management of patients with influenza?  Dickson E.M.; Zambon M.; Pebody R.; de Lusignan S.; Elliot A.J.; Ellis J.; Lackenby A.; Smith G.; McMenamin J.  Eurosurveillance 2020;25(44):1900420  2020  DOI: [10.2807/1560-7917.ES.2020.25.44.1900420](https://dx.doi.org/10.2807/1560-7917.ES.2020.25.44.1900420) | 24/05/23 | HN, JS | Wrong study design | No original research described |
| 6 | Mixed impact of xpert MTB/RIF on tuberculosis diagnosis in Cambodia  Auld S.C.; Moore B.K.; Kyle R.P.; Eng B.; Nong K.; Pevzner E.S.; Eam K.K.; Eang M.T.; Killam W.P.  Public Health Action 2016;6(2):129-135  2016  DOI: [10.5588/pha.16.0001](https://dx.doi.org/10.5588/pha.16.0001) | 24/05/23 | HN, JS | Wrong outcome | No qualitative outcome |
| 7 | Point-of-care virologic testing to improve outcomes of HIV-infected children in Zambia: A clinical trial protoco  Chibwesha C.J.; Ford C.E.; Mollan K.R.; Stringer J.S.A.  J. Acquired Immune Defic. Syndr. 2016;72(Supplement2):S197-S201  2016  DOI: [10.1097/QAI.0000000000001050](https://dx.doi.org/10.1097/QAI.0000000000001050) | 24/05/23 | HN, JS | Wrong outcome | Lacking qualitative data |
| 8 | Barriers to Point-of-Care Testing in India: Results from Qualitative Research across Different Settings, Users and Major Diseases  Engel, N; Ganesh, G; Patil, M; Yellappa, V; Pai, NP; Vadnais, C; Pai, M  PLOS ONE 2015;10(8):  2015  DOI: [10.1371/journal.pone.0135112](https://dx.doi.org/10.1371/journal.pone.0135112) | 10/04/24 | HN, JS | Wrong study design | Exploratory study with a variety of health workers in many setting- no actual implementation of testing – other work from this author and same year included |
| 9 | Field evaluation of three point-of-care tests for chlamydia and gonorrhoea in remote health services in Australia  Causer L.M.; Hengel B.; Natoli L.; Tangey A.; Badman S.; Tabrizi S.N.; Whiley D.; Ward J.; Kaldor J.M.  Sex. Transm. Infect. 2013;89(SUPPL. 1):  2013  DOI: [10.1136/sextrans-2013-051184.0214](https://dx.doi.org/10.1136/sextrans-2013-051184.0214) | 3/06/23 | HN, JS | Wrong outcome | Lacking qualitative data |
| 10 | Performance and implementation evaluation of the Abbott BinaxNOW rapid antigen test in a high-throughput drive-through community testing site in Massachusetts  Pollock N.R.; Jacobs J.R.; Tran K.; Cranston A.E.; Smith S.; O'Kane C.Y.; Roady T.J.; Moran A.; Scarry A.; Carroll M.; Volinsky L.; Perez G.; Patel P.; Gabriel S.; Lennon N.J.; Madoff L.C.; Brown C.; Smole S.C.  J. Clin. Microbiol. 2021;59(5):e00083-21  2021  DOI: [10.1128/JCM.00083-21](https://dx.doi.org/10.1128/JCM.00083-21) | 03/06/23 | HN, JS | Wrong setting | Drive through testing, later reported by phone |
| 11 | Challenges facing TB control in india focusing on the role of Xpert MTB/RIF in the public and private sector  Nathavitharana R.  Am. J. Trop. Med. Hyg. 2014;91(5 SUPPL. 1):167  2014 | 20/09/23 | HN, JS | Insufficient data | No DOI, appears to be a conference abstract |
| 12 | The impact of Xpert MTB/RIF in sparsely populated rural settings  Van Den Handel T.; Hampton K.H.; Sanne I.; Stevens W.; Crous R.; Van Rie A.  Int. J. Tuberc. Lung Dis. 2015;19(4):392-398  2015  DOI: [10.5588/ijtld.14.0653](https://dx.doi.org/10.5588/ijtld.14.0653) | 24/05/23 | HN, JS | Wrong outcomes | No qualitative data |
| 13 | Provider reported barriers and solutions to improve testing among tuberculosis patients 'eligible for drug susceptibility test': A qualitative study from programmatic setting in India  Shewade, HD; Kokane, AM; Singh, AR; Parmar, M; Verma, M; Desikan, P; Khans, SN; Kumar, AMV  PLOS ONE 2018;13(4):  2018  DOI: [10.1371/journal.pone.0196162](https://dx.doi.org/10.1371/journal.pone.0196162) | 03/06/23 | HN, JS | Wrong intervention | No molecular testing implemented at community-level – it appears samples are collected and transported to lab to get tested |
| 14 | ning rates for HBV, HCV, HDV and hepatocellular carcinoma (HCC) and low rates of antiviral therapy in Mongolia: Results from survey of physicians from all major provinces of Mongolia  Le A.K.; Estevez J.; Kim Y.; Israelski D.M.; Baatarkhuu O.; Sarantuya T.; Narantsetseg S.; Nymadawa P.; Le R.H.; Yuen M.-F.; Dusheiko G.; Rizzetto M.; Nguyen M.H.  Gastroenterology 2016;150(4 SUPPL. 1):S252-S253  2016 | 3/06/23 | HN, JS | Wrong study design | No specific community-testing implemented – wide reach phone survey to physicians from major provinces |
| 15 | A survey on use of rapid tests and tuberculosis diagnostic practices by primary health care providers in South Africa: Implications for the development of new point-of-care tests  Davids M.; Dheda K.; Pai N.P.; Cogill D.; Pai M.; Engel N.  PLoS ONE 2015;10(10):e0141453  2015  DOI: [10.1371/journal.pone.0141453](https://dx.doi.org/10.1371/journal.pone.0141453) | 24/05/23 | HN, JS | Wrong setting | No specific testing in community – healthcare workers form two major cities interviewed on use of general rapid tests |
| 16 | Mixed-methods evaluation of point-of-care hepatitis C virus RNA testing in a Scottish prison.  Byrne, Christopher J; Malaguti, Amy; Inglis, Sarah Karen; Dillon, John F  BMJ open / 2023;13(4):e068604  England 2023 /  DOI: [10.1136/bmjopen-2022-068604](https://dx.doi.org/10.1136/bmjopen-2022-068604) | 12/04/24 | HN, JS | Wrong study design | No specific community-based testing implemented -POC in prison |
| 17 | The NSEBA Demonstration Project: implementation of a point-of-care platform for early infant diagnosis of HIV in rural Zambia  Sutcliffe C.G.; Moyo N.; Schue J.L.; Mutanga J.N.; Hamahuwa M.; Munachoonga P.; Maunga S.; Thuma P.E.; Moss W.J.  Trop. Med. Int. Health 2021;26(9):1036-1046  2021  DOI: [10.1111/tmi.13627](https://dx.doi.org/10.1111/tmi.13627) | 03/06/23 | HN, JS | Wrong outcomes | Not enough qualitative measures |
| 18 | Acceptability, feasibility and cost of point of care testing for sexually transmitted infections among South African adolescents where syndromic management is standard of care.  Marcus, Rebecca; C, Pike; Gill, K; Smith, P; Rouhani, S; Mendelsohn, A; Mendel, E; Lince-Deroche, N; Naidoo, K; Ahmed, N; Stirrup, O; Roseleur, J; Leuner, R; Meyer-Rath, G; Bekker, L G  BMC health services research / 2023;23(1):1078  England 2023 /  DOI: [10.1186/s12913-023-10068-8](https://dx.doi.org/10.1186/s12913-023-10068-8) | 10/04/24 | HN, JS | Wrong study design | Observational study – not really looking at implementation factors but acceptability |
| 19 | Operational experiences associated with the implementation of near point-of-care early infant diagnosis of HIV in Myanmar: a qualitative study  Yee W.L.; Htay H.; Mohamed Y.; Nightingale C.E.; Tin H.H.; Thein W.; Kyaw L.L.; Yee W.W.; Aye M.M.; Badman S.G.; Vallely A.J.; Anderson D.; Kelly-Hanku A.; Luchters S.  BMC Health Serv Res 2021;21(1):863  2021  DOI: [10.1186/s12913-021-06797-3](https://dx.doi.org/10.1186/s12913-021-06797-3) | 3/06/23 | HN, JS | Wrong setting | Implementation into public hospitals as part of routine service |
| 20 | Usability of a novel lateral flow assay for the point-of-care detection of Neisseria gonorrhoeae: A qualitative time-series assessment among healthcare workers in South Africa  de Vos L.; Daniels J.; Gebengu A.; Mazzola L.; Gleeson B.; Piton J.; Mdingi M.; Gigi R.; Ferreyra C.; Klausner J.D.; Peters R.P.H.  PLoS ONE / 2023;18(6 June):e0286666  United States Public Library of Science 2023 /  DOI: [10.1371/journal.pone.0286666](https://dx.doi.org/10.1371/journal.pone.0286666) | 15/04/24 | HN, JS, MWK | Wrong intervention | Non-molecular POC tests, using GX as comparator but not actually studying it |
| 21 | Multidisease testing for HIV and TB using the GeneXpert platform: A feasibility study in rural Zimbabwe  Ndlovu Z.; Fajardo E.; Mbofana E.; Maparo T.; Garone D.; Metcalf C.; Bygrave H.; Kao K.; Zinyowera S.  PLoS ONE 2018;13(3):e0193577  2018  DOI: [10.1371/journal.pone.0193577](https://dx.doi.org/10.1371/journal.pone.0193577) | 03/06/23 | HN, JS | Wrong outcomes | No qualitative data |
| 22 | Peripheral clinic versus centralized laboratory-based Xpert MTB/RIF performance: Experience gained from a pragmatic, stepped-wedge trial in Botswana  Agizew T.; Boyd R.; Ndwapi N.; Auld A.; Basotli J.; Nyirenda S.; Tedla Z.; Mathoma A.; Mathebula U.; Lesedi C.; Pals S.; Date A.; Alexander H.; Kuebrich T.; Finlay A.  PLoS ONE 2017;12(8):e0183237  2017  DOI: [10.1371/journal.pone.0183237](https://dx.doi.org/10.1371/journal.pone.0183237) | 25/05/23 | HN, JS | Wrong outcomes | No qualitative data |
| 23 | Perceived feasibility, facilitators and barriers to incorporating point-of-care testing for SARS-CoV-2 into emergency medical services by ambulance service staff: a survey-based approach  Green, K; Micocci, M; Hicks, T; Winter, A; Martin, JE; Shinkins, B; Shaw, L; Price, C; Davies, K; Allen, JA  BMJ OPEN 2022;12(11):  2022  DOI: [10.1136/bmjopen-2022-064038](https://dx.doi.org/10.1136/bmjopen-2022-064038) | 10/04/24 | HN, JS | Wrong study design | Rapid lateral flow SARS-CoV-2 tested implementation in ambulance service in the UK |
| 24 | Implementation of tuberculosis service integration into ANC and PMTCT programs in northern nigeria  Anih G.; Abraham O.; Dunia E.; Sam-Agudu N.; Ekong E.; Ibrahim H.; Dakum P.  J. Acquired Immune Defic. Syndr. 2019;81(Supplement 1):74  2019 | 03/06/23 | HN, JS | Wrong study design | More of an evaluation of a screening sercive, than actualy integration of molecular testing ie “A TB integration manual, and TB screening algorithm and data-capturing tools were developed and deployed to facilities, along with healthcare worker engagement and training.” |
| 25 | Integrating human papillomavirus testing as a point-of care service using GeneXpert platforms: Findings and lessons from a Kenyan pilot study (2019-2020).  Mwenda, Valerian; Bor, Joan-Paula; Nyangasi, Mary; Njeru, James; Olwande, Sharon; Njiri, Patricia; Arbyn, Marc; Weyers, Steven; Tummers, Philippe; Temmerman, Marleen  PloS one / 2023;18(5):e0286202  United States 2023 /  DOI: [10.1371/journal.pone.0286202](https://dx.doi.org/10.1371/journal.pone.0286202) | 15/04/24 | HN, JS, MWK | Wrong population | Tests are being run by laboratory personnel at these hospitals.  Our population of interest is non-laboratory staff doing these tests. |
| 26 | Public health implications of molecular point-of-care testing for chlamydia and gonorrhoea in remote primary care services in Australia: a qualitative study  TTANGO Investigator Grp; Natoli, L; Guy, RJ; Shephard, M; Whiley, D; Tabrizi, SN; Ward, J; Regan, DG; Badman, SG; Anderson, DA; Kaldor, J; Maher, L  BMJ OPEN 2015;5(4):  2015  DOI: [10.1136/bmjopen-2014-006922](https://dx.doi.org/10.1136/bmjopen-2014-006922) | 10/04/24 | HN, JS | Wrong study design | No actual implementation of community-based testing – exploration of public health intervention - did however include a full-text publication from this author group that was part of the same trial (TTANGO) |
| 27 | Implementation and Operational Research: Implementation of Multiple Point-of-Care Testing in 2 HIV Antiretroviral Treatment Clinics in South Africa  Gous N.M.; Scott L.E.; Potgieter J.; Ntabeni L.; Sanne I.; Stevens W.S.  J. Acquired Immune Defic. Syndr. 2016;71(2):e34-e43  2016  DOI: [10.1097/QAI.0000000000000872](https://dx.doi.org/10.1097/QAI.0000000000000872) | 25/05/23 | HN, JS | Wrong outcomes | Not enough qualitative data |
| 28 | A qualitative study on health facility utilization of GeneXpert machines for TB diagnosis in Tanzania  Pamba D.; Kondo Z.; Nkiligi E.; Olomi W.; Sabi I.; Ntinginya N.  Tanzan. J. Health Res. 2022;23(Supplement 1):128  2022  DOI: [10.4314/thrb.v23i1.1S](https://dx.doi.org/10.4314/thrb.v23i1.1S) | 25/03/23 | HN, JS | Insufficient data | Abstract does not contain enough data – no full text |
| 29 | Implementation of Xpert MTB/RIF for routine point-of-care diagnosis of tuberculosis at the primary care level  Clouse K.; Page-Shipp L.; Dansey H.; Moatlhodi B.; Scott L.; Bassett J.; Stevens W.; Sanne I.; Van Rie A.  S. Afr. Med. J. 2012;102(10):805-807  2012  DOI: [10.7196/SAMJ.5851](https://dx.doi.org/10.7196/SAMJ.5851) | 03/06/23 | HN, JS | Wrong outcomes | No qualitative outcomes described ie interviews, survey ect, more of an experience piece? |
| 30 | Point-of-care testing in India: missed opportunities to realize the true potential of point-of-care testing programs  Engel, N; Ganesh, G; Patil, M; Yellappa, V; Vadnais, C; Pai, NP; Pai, M  BMC HEALTH SERVICES RESEARCH 2015;15():  2015  DOI: [10.1186/s12913-015-1223-3](https://dx.doi.org/10.1186/s12913-015-1223-3) | 10/04/24 | HN, JS | Wrong study design | No actual implementation of community-based testing |
| 31 | Caregiver experience and perceived acceptability of a novel near point-of-care early infant HIV diagnostic test among caregivers enrolled in the PMTCT program, Myanmar: A qualitative study  Yee W.L.; Than K.K.; Mohamed Y.; Htay H.; Tin H.H.; Thein W.; Kyaw L.L.; Yee W.W.; Aye M.M.; Badman S.G.; Vallely A.J.; Luchters S.; Kelly-Hanku A.  PLoS ONE 2020;15(10 October):e0241245  2020  DOI: [10.1371/journal.pone.0241245](https://dx.doi.org/10.1371/journal.pone.0241245) | 03/06/23 | HN, JS | Wrong study design | Designed to evaluate caregiver experience, not medical professions or implementation factors |
| 32 | Benefits and barriers to integration of chlamydia and gonorrhea point-of-care testing into remote communities  Natoli L.; Maher L.; Kaldor J.; Ward J.; Shephard M.; Anderson D.; Guy R.  Sex. Transm. Dis. 2014;41(SUPPL. 1):S26  2014 | 20/09/23 | HN, JS | Insufficient data | Conference abstract with insufficient data – did however include a full-text publication from this author group that was part of the same trial (TTANGO) |
