# Supplementary material for: A qualitative systematic review of barriers and facilitators to the implementation of community-based molecular diagnostics for infectious diseases
Source: PLoS One. 2025 May 13;20(5):e0321690. doi: 10.1371/journal.pone.0321690 (PMC12074526; doi:10.1371/journal.pone.0321690)
Supplement: S5 Table — (DOCX) [file pone.0321690.s005.docx]

S5 Table. Included studies data extraction

| **Article** | **Initials of Data Extractors** | **Date of data extraction** | **Country** | **Facility Type** | **Qualitative Data Collection Method** | **Qualitative Focus** | **Participants** | **Sample Size**  **(n = )** | **Testing Implemented** | **Key Qualitative Findings Extracted^a^** |
| --- | --- | --- | --- | --- | --- | --- | --- | --- | --- | --- |
| Martin et al. 2022  *“It was difficult to offer same day results”: evaluation of community-based point-of-care testing for sexually transmitted infections among youth using the GeneXpert platform in Zimbabwe.* | HN, JS | February 2024 | Zimbabwe | Community-care centre | In-depth interviews | Facilitators and barriers to point‐of‐care testing for *Chlamydia trachomatis* (CT) and *Neisseria gonorrhoea* (NG) for youth in community‐based settings in Zimbabwe | Program (CHIEDZA) providers (four CHWs, two nurses, two youth workers, and one counsellor) and  team members involved in the development and co-ordination of on-site testing (the trial co-ordinator, a microbiologist, and a laboratory technician) | 12 | Xpert® CT/NG | The interviews with service providers revealed four themes related to the provision of same day CT/NG results associated with the i) Limitations of the diagnostic device ii) Challenges with implementation in a community setting, iii) Provider buy-in, and iv) Competing priorities for clients.  Despite an increase in testing capacity by replacing the two-module instrument with a four-module instrument, providers noted that sample throughput over the six and a half hours available for testing was insufficient, resulting in a backlog of samples which needed to be tested off-site. As a result, samples were processed the following day or later and so “*it was difficult to offer same day results.”*  Sample runs resulting in an error message were noted to have caused disruption for CHIEDZA services. Providers noted that error rates reduced *“with experience and time”* as they became more confident using the device.  Reported challenges for community setting included electrical supply, high temperatures and weather conditions such as wind and rain. This was particularly challenging at one site, where services were provided in an outside space rather than inside a community centre. Space shortage was also a concern  Training was an important facilitator ensuring that providers were able to use the GeneXpert machine. The training received was generally felt to be adequate and most providers found using the machine *“simple and straightforward”*.  The introduction of on-site CT/NG testing led to an increase in workload for CHIEDZA providers. This included both a higher intensity of work at the sites as providers had to juggle CT/NG testing with other duties, as well as the need for excess samples to be processed outside of nor- mal working hours.  There was a strong perception amongst providers that there was limited demand from clients for same day results, given the time clients had to wait, except in specific limited circumstances.  There was a perception that clients simply *“cannot wait for 90 minutes”* for their results. |
| Mohammad et al. 2020 *Feasibility and acceptability of implementing early infant diagnosis of HIV in Papua New Guinea at the point of care: a qualitative exploration of health worker and key informant perspectives.* | HN, JS | February 2024 | Papua New Guinea | Two provincial hospitals with no laboratory technicians | Semi-structured interviews |  | Nurses and key informant (five senior prevention of mother-to-child transmission [PMTCT] programme staff, two Central Public Health Laboratory staff, three national government representatives, and three NGO staff) | Nurses = 5  Key informants = 13 | Xpert® HIV-1 qualitative | Being able to commence HIV-infected infants on ART ‘straight away’ was identified by all study participants as the main advantage of POC EID testing, reducing the likelihood of child morbidity and mortality. Another benefit of POC EID testing was a sense of job satisfaction at providing good quality care to caregivers and babies.  Health workers, laboratory staff and policymakers in all sites noted the long turnaround time between DBS collection from HIV-exposed infants and the communication of results to facilities and families.  Staff in the study sites stated that more caregivers were coming to the clinics for EID testing as a result of the availability of same-day results than would normally be expected with standard EID testing. Health workers felt that caregivers were more motivated to return to the clinic for appointments in order to find out if their child or children had HIV, and that the majority were willing to wait for 90 min to receive HIV test results.  Research nurses were recruited by the PNG Institute of Medical Research and collocated at both study sites to undertake study procedures and conduct POC EID testing during the intervention phase of the study. In some cases, they also relayed test results to caregivers. According to the health workers interviewed, the presence of these additional staff made implementation feasible at the study sites.  Participants emphasised the importance of training and hiring an appropriate number of staff in facilities where POC EID services would be implemented.  Despite acknowledging the potential for an increased workload, the majority of health workers were willing to spend the time conducting POC EID due to its perceived importance for the quality of care provided. |
| Opollo et al. 2018  *Field evaluation of near point of care Cepheid GeneXpert HIV-1 Qual for early infant diagnosis.* | HN, JS | February 2024 | Kenya | Sub-county hospital (1), health centers (3) and dispensaries (22) for sample collection, with central hubs between them for testing (4) | Self-administered questionnaire | The laboratory technologists using the POC in the field were assessed on their feedback on using the new technology, focusing on the operational characteristics, ease of use and technique of the POC | Laboratory technologists conducting testing in the field | Not reported | Xpert® HIV-1 | Reported the device was easy to use and instructions for both device and test were easy to follow and interpret.  Noted difficulties interpreting the EID resulting graph, a few cases of increased temperatures within the POC devices, a longer run time per test and frequent power fluctuations although batteries were available as power back-up.  Staff reported that the GeneXpert was a high performing device that was easy and feasible to use within the field setting.  Advantages of the device included the use of dried blood spot (DBS) to run the test, limited specimen preparation for DBS, hands-on device with limited user engagement, and ability to test at least 4 samples simultaneously on the different modules with detailed results printout and stored electronically within the POC device. |
| Ardizzoni et al. 2015 *Implementing the Xpert® MTB/RIF Diagnostic Test for Tuberculosis and Rifampicin Resistance: Outcomes and Lessons Learned in 18 Countries.* | HN, JS | February 2024 | 18 LMICs* | District and sub-district laboratories (21), regional facilities (5), peripheral facilities (6) and one penitentiary system facility | Questionnaire | Identify the key lessons learned from implementing the GeneXpert system and Xpert testing from each study site | Site laboratory coordinators | 28 | Xpert® MTB/RIF | Projects reported installation of air conditioning as one of the main logistical interventions (54%), followed by installation of a generator (39%), while the majority of the laboratories were already equipped with a biosafety cabinet prior to Xpert implementation (89%).  High rates of inconclusive results were reported as one of the main limitations by almost half of the respondents. Fourteen respondents mentioned having contacted the manufacturer (Cepheid) specifically regarding the high rate of inconclusive results.  All respondents reported being generally satisfied with the system. However, some commented that discordant results between Xpert and culture made interpretation of results difficult, that bloody sputum resulted in inconclusive results, and that viruses occasionally infected the computer used with the system.  The Xpert system was initially described as easy to perform, requiring minimal training and set up, including in peripheral settings. However, in our experience the device was not uniformly easy to install and operate. Its implementation required costly interventions, including provision of air conditioning, provision of uninterrupted electricity and internet connection for calibration. Until a more robust system is available, these extra costs need to be taken into account prior to the decision to introduce the test. The feedback from users was overall positive, mainly due to the simplicity of the procedure. However, aside from logistical interventions, implementation required regular technical support, including training in results interpretation, which had to be adapted to the level of the facility, such as in the case of reference laboratories due to discordant results with culture techniques. Language issues, which initially hindered implementation in some sites, were eventually addressed by the manufacturer. |
| Engel et al. 2015 *Compounding diagnostic delays: a qualitative study of point-of-care testing in South Africa.* | HN, JS | February 2024 | South Africa | Urban and rural public or private settings, ranging from community/clinic setting to hospital or peripheral lab | Semi-structured interviews | Testing and barriers to POC testing conducted in hospital and clinic settings | Doctors, nurses, community health workers, patients, laboratory technicians, policymakers, hospital managers and diagnostic manufacturers | 101 | Variety of tests for HIV TB including Xpert® MTB/RIF | In those few public clinics that have a Xpert MTB/RIF onsite to test TB, the TAT is usually 24 hours and not 90 min as the device promises, due to the large amount of samples the clinic runs daily. A NHLS consultant highlights that not only were 2.5 additional nurses needed to conduct the test in clinics, the machine also lies idle at night and the promise to have results available within one encounter does not translate to same-day decisions in clinical practice.  However, when tests are conducted on the spot in clinics and hospital wards (with sufficient manpower and equipment), test result-based management decisions (treatment, referral, follow-up testing) are being made in one patient encounter and POC continuums can be ensured. The exception is Xpert MTB/RIF that cannot be made to work in one encounter, because results are only available after 24 h due to backlogs and manpower requirements  Experiences with Xpert MTB/RIF also show how essential it is that tests fit into workflows and human resource capacities at POC. If manpower is insufficient and work- load too high, delays deter the POC continuum. Also, it matters how tests are embedded in follow-up testing schedules; additional testing with long TATs can again delay treatment initiation. These factors are absent in the current literature on health system delays in diagnosing TB and HIV. |
|  |  |  |  |  | Focus group discussions |  | TB patients, nurses, and community health workers | 40 (7 groups) |  |  |
| Natoli et al. 2015  *“I Do Feel Like a Scientist at Times”: A Qualitative Study of the Acceptability of Molecular Point-Of-Care Testing for Chlamydia and Gonorrhoea to Primary Care Professionals in a Remote High STI Burden Setting.* | HN, JS | February 2024 | Australia | Aboriginal community-controlled health services in remote communities (12) | In-depth interviews | Explore the acceptability of the GeneXpert to primary care staff in remote Australia | Registered or enrolled nurses and Aboriginal Health Workers/Practitioners | 16 | Xpert® CT/NG | **Attitudes**  Staff empowerment: Having access to a point-of-care device empowered staff, as they felt they were responsible for new technology which could improve the health of people in the community. This was particularly true for Aboriginal Health Practitioners/Workers.  Job satisfaction: Given the delays associated with receiving results from laboratories in remote communities, and the challenges in locating clients for treatment when positive results are returned, participants commonly reported high levels of satisfaction with being able to test and treat on the same day.  Increased awareness of STIs: Approximately half of the participants commented that the presence of the point-of-care device led to heightened awareness of STIs and testing, but were unsure if this would be sustained.  Enables more comprehensive care: Some staff commented on how the point-of-care test approach (and the 90 minute wait for results) created the space for broader health education and also gave an opportunity to invite clients to take part in a complete adult health check [an Australian government initiative that targets Aboriginal people, 15–54 years, to facilitate early detection, diagnosis and management of common, treatable conditions].  **Usefulness/Utility**  Reducing loss to follow up: Many of the participants spoke of the significant population mobility in remote communities, and how delays associated with traditional laboratory testing meant that clients with positive results could not always be located for treatment. Participants felt that the point-of-care approach overcame this challenge.  Reducing recall efforts: Delays in result turn-around associated with routine laboratory testing mean that clients with positive results need to be recalled to the clinic for treatment. Recall processes vary but are inevitably time consuming. Point-of-care testing virtually eliminates the need for client recall associated with routine laboratory testing, where clients are followed up days/weeks after specimen collection when laboratory results become available.  Reducing time to treatment: For many participants, one of the overriding advantages of point-of-care testing was reducing the time between specimen collection and treatment provision. This was seen as particularly important in the context of high client mobility in many remote communities.  **Ease of use**  Sample collection: Respondents liked the fact that the point-of-care device could process both urine and swab specimens (the specimen type collected varied by site according to local guidelines).  Operation of point-of-care device: Most participants found the GeneXpert easy to use and reliable. However, several reported challenges in initially understanding which information was required to be entered in each field on the ‘start a test’ template on the computer- in particular the sample ID field. Some found the data entry process time consuming and suggested that this would be greatly simplified if the machine was linked to the patient management system and these data fields could self-populate.  Connectivity: Several participants commented that the process of documentation (transcribing test results from the GeneXpert into patient notes) was time consuming. This seemed to be exacerbated if the point-of-care device and the computer being used to access patient files were located in a different room.  **Barriers and Enablers**  Confidence in test: Participants generally expressed high levels of confidence in point-of- care test results and were happy to treat patients on this basis. However, a few participants expressed concern about the potential for false positive results, based on a perceived risk of cross contamination from surrounding surfaces and circulating dust during sample preparation.  Impact on workflow: At many health services, clients often left the clinic after the consultation while waiting for the results of the point-of-care test, with staff arranging to call them later in the day with results. At some health services clients were still in the clinic at the time results became available. Either way participants generally reported that the point-of-care testing was minimally disruptive. The exception to this was feedback from staff in very busy/ understaffed services, who felt that the point-of-care approach created ‘another thing’ to remember and track in an already busy environment.  Acceptability to clients: When reflecting on their clients’ experience of point-of-care test- ing, more than half of the participants reported a high degree of acceptability. Participants thought that their clients were generally pleased to receive their test results more quickly.  Materials and waste: One participant, admittedly from a very small clinic, remarked about the space and storage (air conditioned) requirements for test kits.  Monthly quality control (QC) and quality assurance (QA) testing: The need for staff to perform quality control testing once per month and to test a panel of four external quality assurance swabs twice per year was seen by some as an important task to ensure the point-of- care test was performing well, rather than a burden. However, some admitted to forgetting to do QC testing every month. |

*Cambodia, Central African Republic, Colombia, Democratic Republic of Congo, Georgia, India, Kenya, Kyrgyzstan, Lesotho, Malawi, Mozambique, Myanmar, Russia, Somalia, South Africa, Swaziland, Uzbekistan, Zimbabwe

^a^Results pulled directly from included manuscripts, with minimal paraphrasing
