# Supplementary material for: A qualitative systematic review of barriers and facilitators to the implementation of community-based molecular diagnostics for infectious diseases
Source: PLoS One. 2025 May 13;20(5):e0321690. doi: 10.1371/journal.pone.0321690 (PMC12074526; doi:10.1371/journal.pone.0321690)
Supplement: S1 Fig — (PDF) [file pone.0321690.s006.pdf]

A Qualitative systematic Review of the Barriers and Facilitators to the Implementation of Community-Based Diagnostic Testing for Infectious Diseases

Key Findings

Main Themes

Infastructure

Difficulty provisioning physical space for testing, maintaining sufficient internet connection and electricity, setting up air conditioning for temperature regulation, and maintaining a supply of consumable test cartridges

Usability

The GeneXpert® device was generally praised for being straightforward and feasible to use in field settings, but concerns included its sensitivity to user errors, need for maintenance, limited testing capacity, and high rates of inconclusive results depending on operator experience.

Staffing

Effective training and support improved staff confidence and attitudes toward point-of-care testing, despite challenges with increased workload and integrating testing into existing duties.

Minor Themes

Impact on Treatment

The ability of community-based testing to reduce treatment delays, enabling immediate care, was particularly beneficial for HIV-positive infants and remote communities.

Community Acceptance

Community acceptance of testing varied, with good acceptance in some cases (e.g., STI testing in Australia) but reluctance in others, particularly among youth and families balancing competing priorities during testing

These findings are based on a rigorous review of 6 studies, but there is a notable lack of First Nation specific studies. There is a need to implement and uplift work that addresses the diagnostic service barriers faced by First Nations peoples, as well as consider the systemic factors that contribute to health inequities.

UBC

First Nations Health Authority
Health through wellness

BC Children's Hospital
Research Institute
